# Case report: Congenital Bednar tumor with pregnancy-associated growth and pain: a diagnostic pitfall with blue nevus-like features

**DOI:** 10.3389/fonc.2026.1792949

**Published:** 2026-03-12

**Authors:** Yan Chen, Ying Shi

**Affiliations:** Department of Dermatology, Renmin Hospital of Wuhan University, Wuhan, China

**Keywords:** Bednar tumor, blue nevus, CD34, congenital pigmented lesion, dermatofibrosarcoma protuberans, pain, pregnancy-associated growth

## Abstract

Bednar tumors are rare pigmented variants of dermatofibrosarcoma protuberans (DFSP), accounting for less than 5% of DFSP cases. We report a rare case of a congenitally pigmented lesion that progressively enlarged during pregnancy and developed pain. A 32-year-old woman presented with a blue-black nodule on her right shoulder, which had been present since birth and was initially misdiagnosed as a blue nevus. Histopathological analysis revealed spindle cell proliferation in a storiform pattern with focal melanin pigment. Immunohistochemistry showed strong CD34 positivity and negative S100 staining, consistent with Bednar tumors. This case highlights the importance of considering Bednar tumors in the differential diagnosis of congenital pigmented lesions, particularly those showing interval growth or symptoms.

## Introduction

Bednar tumors are pigmented variants of dermatofibrosarcoma protuberans (DFSP), characterized histologically by spindle cells containing melanin granules. Although DFSP typically manifests as a slowly enlarging, painless dermal nodule in early adulthood, Bednar tumors are rare, and congenital cases are exceedingly uncommon. The pigmented nature of this tumor may clinically resemble blue nevus or melanoma, posing a diagnostic challenge. We present an unusual case of a congenital Bednar tumor that grew during pregnancy and later became painful, a combination of features not previously reported together in the literature.

## Case report

A 32-year-old Chinese woman presented to our dermatology clinic in August 2025 with a dome-shaped, pigmented skin lesion on her right shoulder. The lesion had been present since birth as a small, flat, blue-black macule. Approximately three years before the pregnancy, the lesion had gradually enlarged and eventually increased in size. Six months before her clinical visit, she reported intermittent pain, particularly when the area was subjected to pressure. There was no history of bleeding, ulceration, or trauma to the area. The patient denied systemic symptoms such as fever, weight loss, or lymphadenopathy.

**Figure 1:**
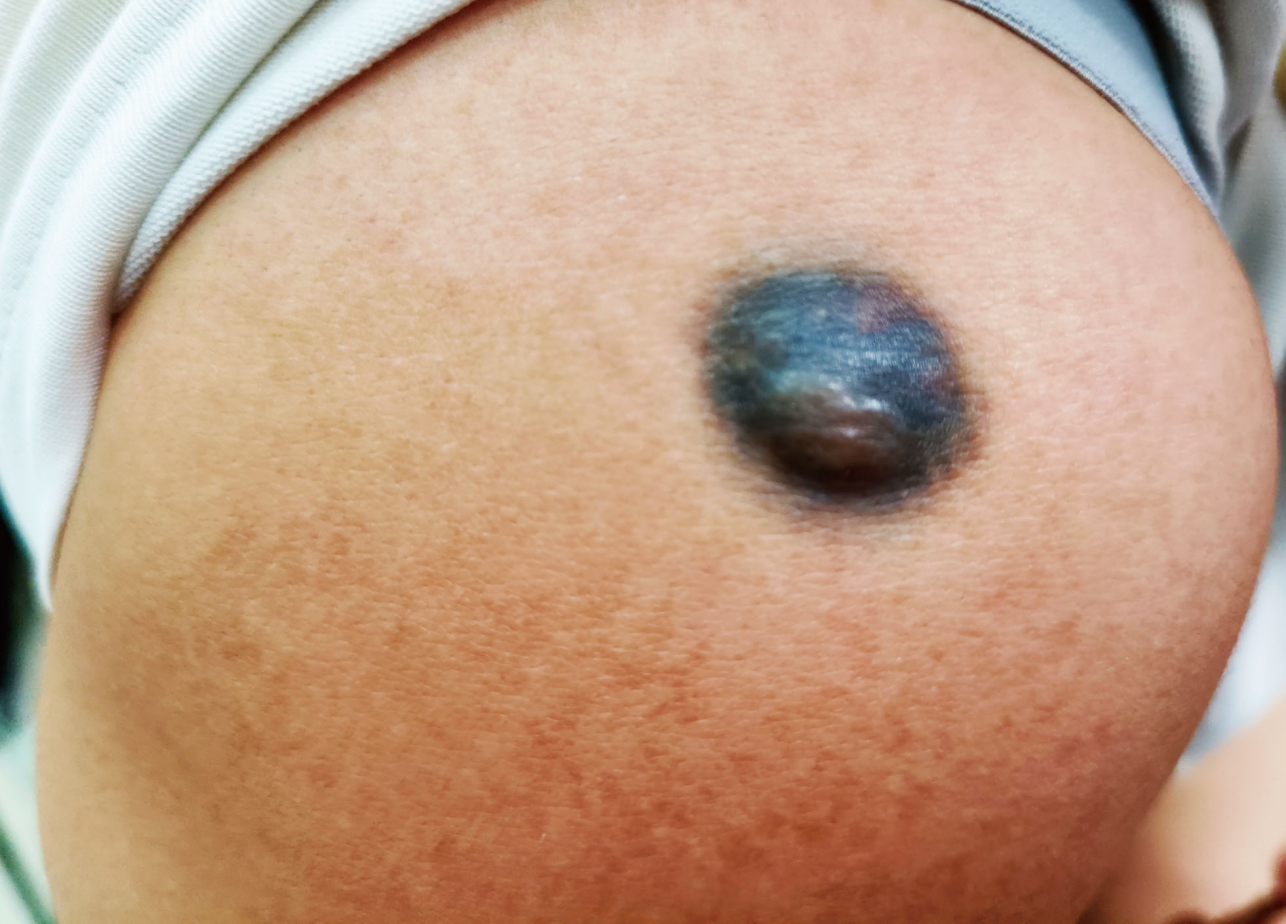
Clinical appearance: A 20mm×15mm bluish-black pigmented nodule on the right shoulder of a 32-year-old woman. The lesion originated as a congenital macule, remained stable for decades, and enlarged markedly during pregnancy.

Physical examination revealed a well-demarcated, dome-shaped, blue-black nodule measuring approximately 20 mm × 15 mm in diameter (**Figure 1**). The lesion was firm, non-mobile, and fixed to the underlying tissue. The overlying skin was intact and smooth. No regional lymphadenopathy was noted.

Due to recent growth and the development of pain, an excisional biopsy was performed under local anesthesia. Gross examination revealed a bluish firm nodule extending into the subcutaneous fat.

**Figure 2:**
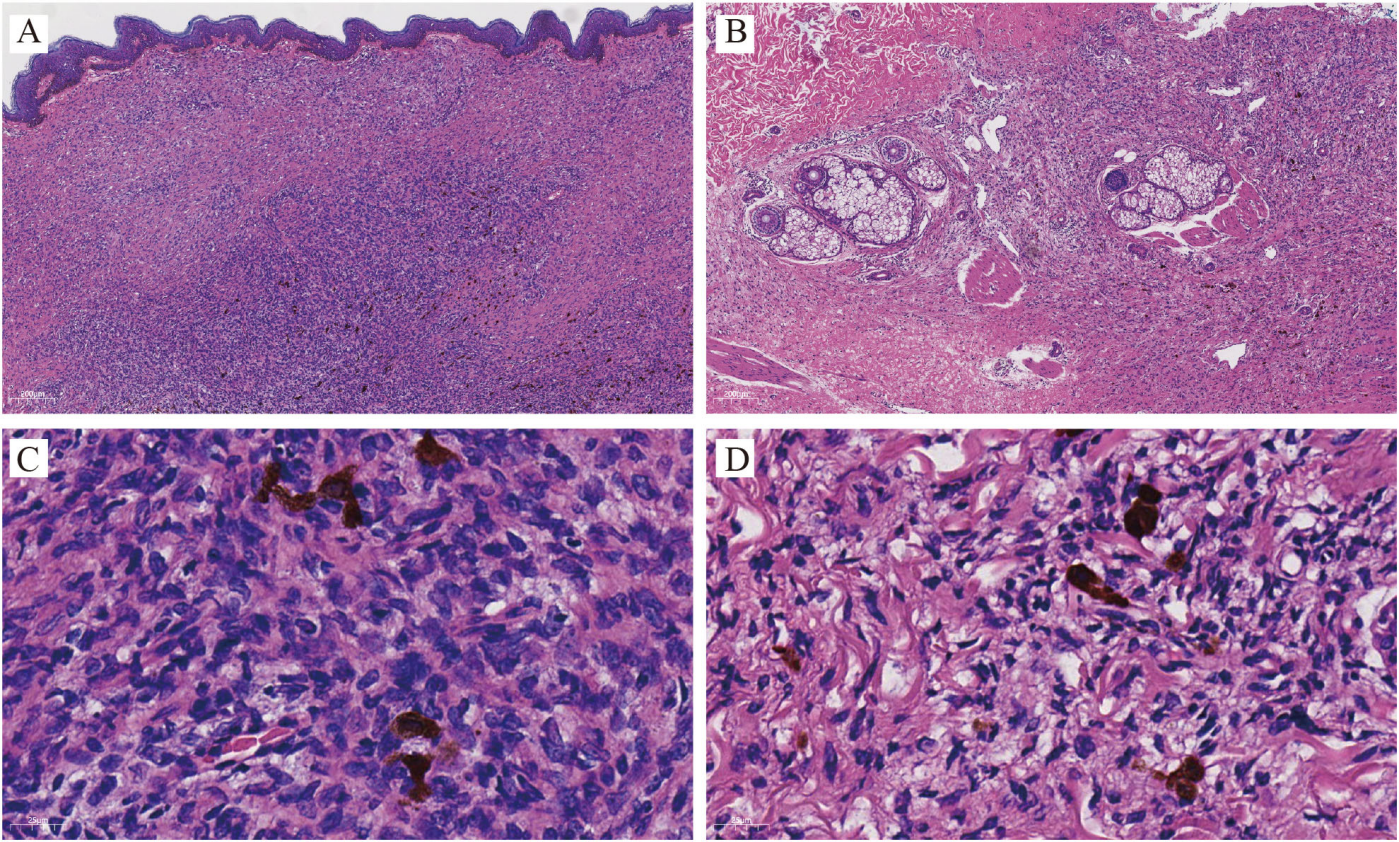
Histopathology (Hematoxylin–eosin): Sections show a storiform proliferation of spindle-shaped tumor cells infiltrating the dermis **(A**, ×40**)** and subcutaneous tissue **(B**, ×40**)**. Basal layer pigmentation of the overlying epidermis is also evident. Higher magnification (×400) reveals spindle cells arranged in interwoven fascicles, with scattered melanin-containing cells. **(C)** Dermis; **(D)** Subcutaneous tissue.

Histopathological evaluation of the excised tissue revealed a spindle cell neoplasm arranged in a storiform pattern extending from the dermis into the subcutaneous adipose tissue. Scattered tumor cells contained brownish melanin granules. No necrosis or abnormal mitotic figures were observed (**Figure 2**).

**Figure 3:**
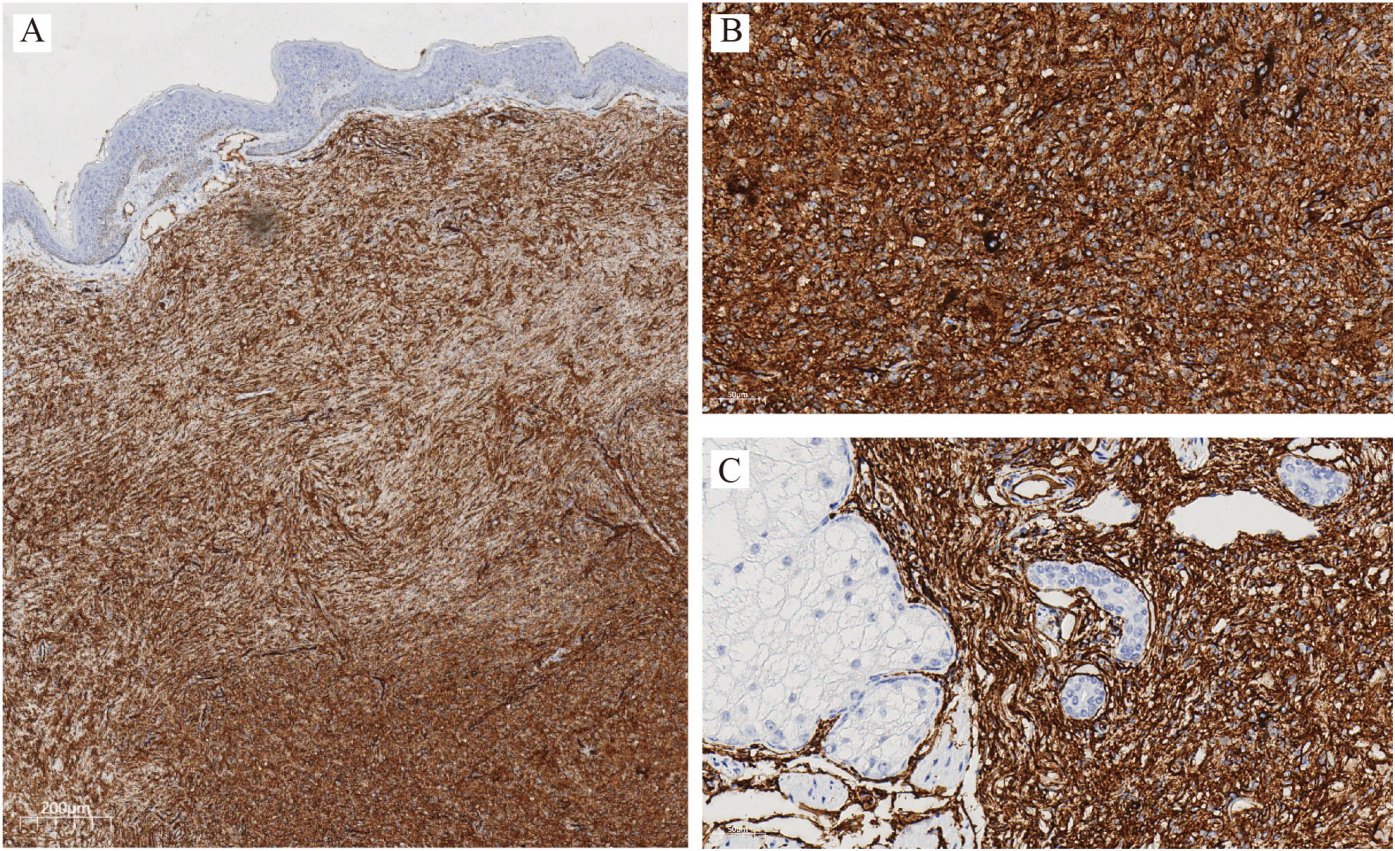
Immunohistochemistry for CD34: Tumor cells show diffuse and strong membranous positivity for CD34, confirming the diagnosis of dermatofibrosarcoma protuberans. **(A)** original magnification ×40; **(B**, ×200**)** Dermis; **(C**, ×200**)** Subcutaneous tissue.

**Figure 4:**
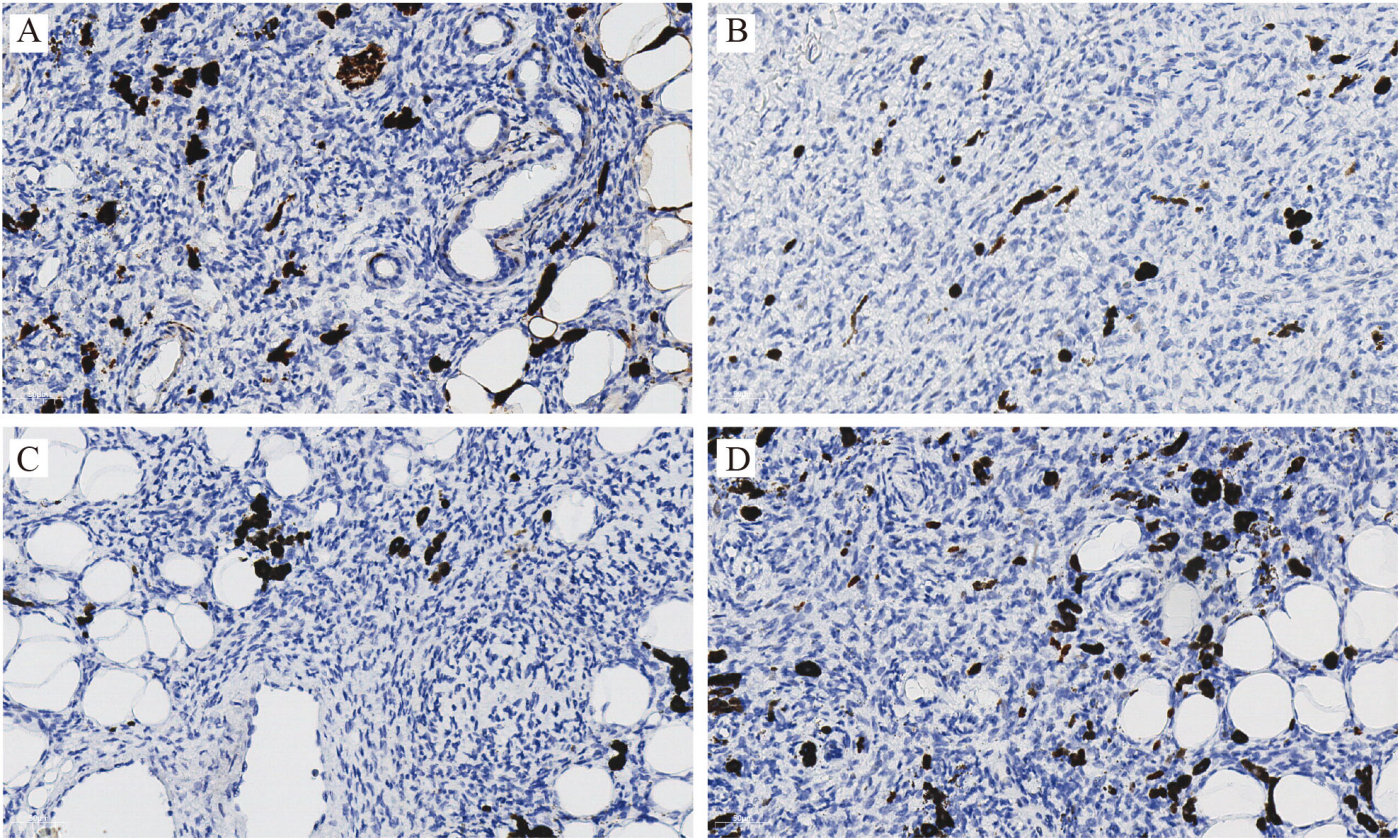
Immunohistochemistry staining: original magnification ×200, S100 **(A)**, SOX10 **(B)**, TRK **(C)** and Ki67 **(D)** all negative staining.

Immunohistochemistry staining showed diffuse and strong CD34 positivity in tumor cells (**Figure 3**). Stains for S100, SOX10, and TRK were negative, ruling out melanocytic or neural origin. The Ki-67 labeling index was low (<5%) (**Figure 4**).

These findings were consistent with a diagnosis of pigmented dermatofibrosarcoma protuberans (Bednar tumor), a rare variant of DFSP. The patient underwent a wide local excision with 3 cm surgical margins. She is currently under follow-up for 6 months with no signs of recurrence to date.

## Discussion

Pigmented DFSP, or Bednar tumor, is a rare pigmented variant of DFSP, first described by Bednar in 1957 (1). It accounts for fewer than 5% of the DFSP cases (2). The presence of melanin-containing dendritic cells and the storiform proliferation of CD34-positive spindle cells characterize this variant (3). Recent studies have shown that Bednar tumor, as a pigmented variant of DFSP, harbors the same characteristic COL1A1–PDGFB fusion gene caused by the reciprocal translocation t(17;22)(q22;q13) (4). This fusion results in constitutive activation of the platelet-derived growth factor B (PDGFB) receptor tyrosine kinase pathway, driving tumorigenesis (5).

Fluorescence *in situ* hybridization (FISH) and reverse transcription PCR (RT-PCR) have been widely used to detect this rearrangement. Significantly, the presence of melanin-producing dendritic cells in Bednar tumor does not alter the underlying genetic hallmark shared with conventional DFSP (6). The identification of this fusion has not only diagnostic value but also therapeutic relevance, as tumors with a COL1A1–PDGFB fusion are known to be sensitive to imatinib mesylate. This tyrosine kinase inhibitor targets PDGFRβ. This highlights the importance of considering molecular testing in cases that are diagnostically challenging or in recurrent and metastatic disease. Molecular testing for the COL1A1-PDGFB fusion was discussed with the patient, but she declined to proceed due to the high cost of the test. Therefore, this analysis was not performed, which is acknowledged as a minor limitation of this report.

While DFSP typically arises in early adulthood as a slow-growing, painless tumor, its congenital onset is exceedingly rare and poorly documented in the literature (7).Clinically, pigmented DFSP may mimic several melanocytic and nonmelanocytic lesions. Our patient’s lesion was initially misdiagnosed as a blue nevus due to its longstanding blue-black appearance and superficial localization. However, the lesion’s subsequent enlargement and pain warrant re-evaluation.

Differential diagnoses for blue–black cutaneous nodules include blue nevus, melanoma, and pigmented basal cell carcinoma (BCC) (**Supplementary Table 1**). The accurate diagnosis of Bednar tumors requires histopathological evaluation, supplemented by supportive immunohistochemistry. CD34 positivity and S100/SOX10 negativity are distinguishing features of Bednar tumor. Although rare, S100 expression may occur in DFSP, often linked to fibrosarcomatous transformation and head-neck location. Such co-expression of CD34 and S100, in the absence of SOX10, resembles the profile of *Neurotrophic Tyrosine Receptor Kinase (NTRK)*-rearranged spindle cell tumors (6).

The key novelty of this case is the combination of congenital onset, pregnancy-associated enlargement, and pain. To highlight the uniqueness of this presentation, we summarized prior reports for each feature separately in **Table 1**.

**Table 1:** Comparison of clinical features in Bednar tumor and DFSP: current case vs. Literature.

Feature	Current case	Literature on Bednar tumor	Literature on conventional DFSP	References
Congenital Onset	Yes	Very rare	Rare (~6% of cases)	(7) (8) (9) (2),,,
Pregnancy-Associated Growth	Yes	No specific reports found	Uncommon (~18 cases by 2021)	(10) (11) (12),,
Pain Development	Yes	Rare (2/6 cases in one series)	Uncommon (~15% of cases)	(13) (14),

Pain is an atypical symptom of DFSP or Bednar tumors, which are commonly asymptomatic (13). In our case, the onset of pain may have been attributable to local nerve compression or inflammation within a confined dermal space. While pain is reported in approximately 15% of conventional DFSP cases, it is considered even rarer in Bednar tumors, with one early series noting occasional pain in only two of six patients (14).

This report contributes to the limited literature on Bednar tumors and highlights the importance of biopsy for long-standing or changing pigmented lesions. Interestingly, the lesion begins to enlarge during pregnancy. Although the exact mechanism is unclear, it is hypothesized that hormonal changes during pregnancy, including elevated levels of estrogen, progesterone, and placental growth factors, may contribute to tumor progression (10). Such pregnancy-associated growth has been documented in other soft tissue tumors, such as neurofibromas (15) and meningiomas (16), but remains poorly described in DFSP variants. While accelerated growth during pregnancy has been documented in a small number of conventional DFSP cases (10, 11), to our knowledge, this is the first report of such a phenomenon in a Bednar tumor.

## Conclusion

Histologically, Bednar tumors must be distinguished from pigmented melanocytic lesions. Immunohistochemical staining is crucial: CD34 positivity supports DFSP, whereas melanocytic markers, such as S100, SOX10, and HMB-45, are typically negative. This pattern was consistent in our patient, confirming the diagnosis despite the clinical mimicry of a blue nevus.

We report a case of a congenital Bednar tumor exhibiting the rare combination of pregnancy-related growth and painful symptoms. This case highlights the diagnostic challenges associated with pigmented skin tumors and supports the role of biopsy in evaluating lesions with atypical progression. This case underscores the significance of histological evaluation for atypical or evolving pigmented lesions, particularly in cases with congenital onset or interval changes during hormonal fluctuations. To our knowledge, this is the first report of a congenital Bednar tumor presenting with pregnancy-associated enlargement and pain.

## Data Availability

The original contributions presented in the study are included in the article/supplementary material. Further inquiries can be directed to the corresponding author.
