# Supplementary material for: Case report: Congenital Bednar tumor with pregnancy-associated growth and pain: a diagnostic pitfall with blue nevus-like features
Source: Front Oncol. 2026 Mar 12;16:1792949. doi: 10.3389/fonc.2026.1792949 (PMC13017353; doi:10.3389/fonc.2026.1792949)
Supplement: Supplementary file 1 [file Table1.docx]

**Table 1.** Comparison of clinical and immunohistochemical features among blue nevi, melanoma, pigmented BCC and the classic Bednar tumor

| **Feature** | **Blue nevus** | **Malignant melanoma** | **Pigmented BCC** | **Bednar tumor (classic)** |
| --- | --- | --- | --- | --- |
| **Onset age** | Congenital or young adult | Adults/elderly, sun-exposed | Middle-aged/elderly, often head/neck | Early to middle adulthood (rarely congenital) |
| **Clinical color** | Uniform blue-black | Black, variegated (brown/black/gray) | Brown to blue-black | Blue to black nodule |
| **Growth pattern** | Stable, slow growth | Rapid growth, ulceration/bleeding | Slow growth may ulcerate | Slow, infiltrative growth, locally aggressive |
| **Symptoms** | Asymptomatic | Pain, bleeding, and ulceration are possible | Itching, ulceration possible | Usually asymptomatic (rarely painful) |
| **Histology** | Dermal dendritic melanocytes, well circumscribed | Atypical melanocytes, mitoses, invasion | Basaloid nests with palisading, pigmentation | Spindle cells in a storiform pattern, melanin-laden dendritic cells |
| **CD34** | Negative | Negative | Negative | Positive |
| **S100** | Positive | Positive | Negative or focally weak | Negative (except scattered dendritic melanocytes) |
| **SOX10** | Positive | Positive | Negative | Negative |
| **HMB-45** | Positive | Positive | Negative | Negative |
| **Ki-67** | Low | High | Intermediate | Low |
